# Treating Trauma- Evaluation of a multi-disciplinary psychiatry service for patients post major trauma

**DOI:** 10.1192/j.eurpsy.2023.1104

**Published:** 2023-07-19

**Authors:** G. Crudden, K. Corrigan, C. Smith, Á. Richards, A. M. Doherty, A. M. Clarke

**Affiliations:** 1 Psychiatry, School of Medicine & Medical Science; Department of Psychiatry, UCD; Mater Misericordiae University Hospital; 2 Department of Psychiatry, Mater Misericordiae University Hospital, Dublin, Ireland

## Abstract

**Introduction:**

Research has shown 30-40 % of people who have experienced traumatic injury are at risk of developing mental illness. Some injuries may be the result of mental ill-health, including self-inflicted injury. Furthermore, the development of psychopathology after injury appears to be a major determinant of long term disability. Early intervention can reduce symptom severity and prevent development of mental illness.

Ireland’s National Trauma System Implementation Programme, announced in April 2021, highlights the need for screening for mental disorders.

The Mater Misericordiae University Hospital (MMUH) is designated as one of two national Major Trauma Centres in Ireland. Its trauma service will expand with an expectation of an additional 450- 500 major trauma patients over the next three years.

The Consultation Liaison Psychiatry Service (CLP) currently provides expert mental health input to medical and surgical teams, in managing a range of patients with mental illnesses or psychological difficulties, including those with experience of major trauma.

**Objectives:**

To examine the current mental health service provision for trauma patients over a six-month period. We aimed to identify areas of need to inform future development of a psychiatry-led MDT service for trauma patients.

**Methods:**

A review of all patients admitted on the MMUH trauma pathway between January 2021 and June 2021 was performed. The following data were recorded: demographics, mechanism of injury and information on referrals to the liaison psychiatry service.

**Results:**

There were 105 trauma cases over the six-month period; 46 females and 59 males. The mean age was 58.4 years (SD 22.16). Twelve individuals were recorded as ‘No Fixed Abode’ or living in homeless accommodation(11.4%).

In terms of mechanism of injury; 20 were assaulted of which 8 were stabbing/ knife injuries. There were 65 falls and 12 road traffic accidents. In 3 cases (2.8%), the mechanism of injury was self-inflicted. Twenty patients were admitted to critical care (19%).

Of the 105 trauma patients, 19 (18%) were referred to CLP service; 2 (10.5%) were seen in the outpatient setting, the rest as inpatients (89.5%). At least one repeat review was indicated in 10 of the 19 patients (52.6%).

**Conclusions:**

Trauma patients have a high rate of comorbid mental illness. Nearly 1/5 are currently referred to the CLP service, which is likely an underestimation of the actual burden of mental health disorders and could be explained by the lack of dedicated services. The liaison psychiatry team provides valuable input into the multidisciplinary care of trauma patients and the demand for its services is likely to increase with the expansion under the Major Trauma Strategy for Ireland.

**Disclosure of Interest:**

None Declared

